# BlockSIEM: Protecting Smart City Services through a Blockchain-based and Distributed SIEM

**DOI:** 10.3390/s20164636

**Published:** 2020-08-18

**Authors:** Juan Velandia Botello, Andrés Pardo Mesa, Fabián Ardila Rodríguez, Daniel Díaz-López, Pantaleone Nespoli, Félix Gómez Mármol

**Affiliations:** 1 Escuela Colombiana de Ingeniería Julio Garavito, Bogotá 111166, Colombia; andres.pardo-m@mail.escuelaing.edu.co (A.P.M.); fabian.ardila@mail.escuelaing.edu.co (F.A.R.); 2 Universidad del Rosario, School of Engineering, Science and Technology, Bogotá, 111711, Colombia; 3 Faculty of Computer Science, University of Murcia, 30100 Murcia, Spain; pantaleone.nespoli@um.es (P.N.); felixgm@um.es (F.G.M.)

**Keywords:** smart city, IoT security, blockchain, SIEM, intrusion detection system

## Abstract

The Internet of Things (IoT) paradigm has revolutionized several industries (e.g., manufacturing, health, transport, education, among others) by allowing objects to connect to the Internet and, thus, enabling a variety of novel applications. In this sense, IoT devices have become an essential component of smart cities, allowing many novel and useful services, but, at the same time, bringing numerous cybersecurity threats. The paper at hand proposes BlockSIEM, a blockchain-based and distributed Security Information and Event Management (SIEM) solution framework for the protection of the aforementioned smart city services. The proposed SIEM relies on blockchain technology to securely store and access security events. Such security events are generated by IoT sentinels that are in charge of shielding groups of IoT devices. The IoT sentinels may be deployed in smart city scenarios, such as smart hospitals, smart transport systems, smart airports, among others, ensuring a satisfactory level of protection. The blockchain guarantees the non-repudiation and traceability of the registry of security events due to its features. To demonstrate the feasibility of the proposed approach, our proposal is implemented using Ethereum and validated through different use cases and experiments.

## 1. Introduction

A smart city aims to solve challenges that are caused by population growth in urban areas, e.g., physical security, residual management, and transportation systems, using technology to interconnect governmental organizations and allowing citizens access to multiple services, which supports a way to efficiently and transparently manage different public resources [1]. The Internet of Things (IoT) technology offers low cost and effective solutions for the development of smart cities due to its high applicability in an uncountable number of scenarios [2,3]. In particular, the intelligent IoT devices can continuously collaborate in various sectors of a smart city scenario, exchanging a constant flow of information to offer quality services to citizens as an ultimate goal [4,5]. We refer to the IoT devices as intelligent, in the sense that they are able to autonomously communicate, requiring little or no human intervention [6].

While IoT devices have been able to provide uncountable useful services, the popularization of IoT solutions imposes several security risks, given the lack of supervision and the limitation of computational resources in IoT devices [7]. Several works have focused their attention on discovering the weaknesses of IoT ecosystems, and, consequently, proposing effective solutions to address them [8].

In this sense, as an IoT environment incorporates more devices and components, its attack surface increases, augmenting the possibility of suffering an attack. Particularly, one of the significant risks of disclosing a vulnerable IoT ecosystem is the possibility to expose a large number of IoT devices to infection, which may become members of a botnet capable to execute a distributed attack. A clear example of this situation can be described by looking at the Mirai Distributed Denial of Service (DDoS) attack that infected around 50K IoT devices in 164 different countries, and then used those infected devices to launch further DDoS attacks [9]. In particular, Mirai malware spreads by first infecting IoT devices, such as webcams, Digital Video Recorders (DVRs), and routers. It then guesses the admin credentials of other IoT devices employing brute force, relying on a small dictionary of potential username–password pairs [10].

To solve the security challenges presented by an IoT ecosystem, BlockSIEM proposes a blockchain-based and distributed Security Information and Event Management (SIEM) framework to detect, store, and analyze IoT security events in a trustworthy way, while maintaining their integrity and non-repudiation attributes [11]. In this sense, thanks to blockchain technology, BlockSIEM features several security properties, being non-repudiation, and traceability among the most notable ones. On the one hand, non-repudiation means that the blocks are immutable, and an entity cannot deny that it generated a transaction request, while the traceability of the information recorded in the blocks allows one to know the trail of a specific security event. In addition, BlockSIEM can be deployed in different use cases scenarios, guaranteeing the resilience, trustworthiness, auditability, and scalability of the system. This paper is an extension of [12], where BSIEM-IoT, a blockchain-based SIEM solution that was suitable for IoT environments, was proposed, generating security events thanks to the effort of IoT devices called Sentinels, and reporting these through blockchain to be read and analyzed by SIEM miners having internal and external threat intelligence capabilities.

The main contributions of [12] were the initial proposal of BSIEM-IoT as a blockchain-based SIEM, enclosing a smart contract used to handle blocks of security events to prevent and detect attacks over IoT scenarios. BSIEM-IoT used external and internal Threat Intelligence with the aim of detecting distributed attacks. The evaluation of one use case was also included in [12] as an initial validation of the viability of BSIEM-IoT.

In the paper at hand, the following additional contributions are provided:

- An extension of the state of the art, including a deeper analysis of similar solutions, arguing their pros and cons.
- An adaptation of the architecture of BSIEM-IoT to make it applicable to smart cities scenarios.
- The addition of the capacity of BSIEM-IoT of handling IoT security events, according to the priority and the event types, to guarantee security awareness about the state of the IoT devices being protected and the potential threats detected from the security events.
- An extension of the applicability of BSIEM-IoT to smart cities environments that are composed of different IoT scenarios with features of serverless operations, integrity, non-repudiation, and resiliency. This includes a deeper description of use cases.
- The implementation of several use cases, such as the detection of distributed attacks, the ability of BSIEM-IoT to continue working under hostile conditions, the BSIEM-IoT capabilities to audit security incidents and scale almost effortlessly.
- The above-mentioned use cases were evaluated through exhaustive experiments, which in turn confirmed, and proved the feasibility of the presented solution in real use case scenarios.

The remainder of this paper is structured, as follows: a brief explanation of the main concepts surrounding the blockchain technology is given in Section 2, while Section 3 analyzes some of the key works proposed in the field, highlighting their pros and cons. In Section 4, the novel blockchain-based and distributed SIEM architecture is presented. Next, Section 5 shows several operational use cases describing, in detail, the capabilities of BlockSIEM. Subsequently, in Section 6, we demonstrate the feasibility of employing the innovative proposal through exhaustive experiments. Finally, Section 7 concludes the work, summarizing the main results and highlighting future research directions.

## 2. Background

Blockchain is a decentralized P2P network where all generated transactions are validated by the registered nodes and recorded in a distributed and immutable ledger [13]. In this context, the consensus algorithm is the core of the blockchain technology, as it guarantees the reliability of the network. Specifically, as no central authority is present to verify the produced events, each transaction needs to be validated by the blockchain nodes through a mutual agreement (i.e., consensus) [14]. In the following, some of the most common consensus types are presented [15]:

- Proof of Work (PoW): a transaction is approved if at least half plus one of the nodes accept it in the P2P network.
- Proof of Stake (PoS): the node who has more wealth has greater probability to participate in the consensus and create a block.
- Proof of Importance (PoI): the nodes that can create a block are the ones with the greatest number of transactions in the network.
- Proof of Authority (PoA): only some nodes are explicitly allowed to create new blocks and secure the blockchain.

It has to be perceived that each of the above-mentioned algorithms features possible advantages and potential drawbacks, mainly depending on the underlying P2P network structure [16]. Explicitly, PoW and PoS can be depicted as the most used algorithms to reach the consensus among the P2P nodes. Nevertheless, it has been demonstrated that PoW requires higher computational resources, while PoS is more exposed to attacks, since the mining cost is nearly zero [15]. PoI and PoA represent two valid alternatives, as they are energy-friendly and have a better performance. However, their merits have been questioned, especially in terms of availability and consistency [17,18]. In the context of this research, our proposal leverages the capabilities of the PoW implemented by the Ethereum protocol, as we will see later.

Furthermore, blockchain proposes two ways to build a network [19], namely, permissioned and permissionless blockchains, the main difference being the level of governance of each node. In particular, permissionless blockchains (i.e., public blockchains) allow any possible candidate to become a node and belong to the network. Nodes on this blockchain can perform any task as long as they possess the physical capability (e.g., mine blocks, validate transactions, etc.). In turn, permissioned blockchains (i.e., private blockchains) restrict the access of the nodes that belong to the network and performing tasks. A relevant feature of this kind of blockchain is that it is possible to choose the level of decentralization on the network, i.e., fully centralized or partially decentralized [20]. Concretely, open permissioned blockchains are partially decentralized, since each entity can read the stored data, while closed permissioned blockchains are fully centralized since the stored data are only visible to the participant nodes [21].

By leveraging blockchain technology, one can develop Decentralized Applications or DApps [22], which is, an application which is able to run on a distributed network in a trustworthy fashion. To do so, a Dapp requires a back-end component to correctly assist the transactions and, in this regard, blockchains implement smart contracts (SC) to support any operation required by the application logic. In this direction, Ethereum [23] is an example of an open-source platform to create and manage smart contracts. As we will see later with further detail, BlockSIEM is a closed permissioned blockchain composed by selected nodes supporting a DApp for the management of IoT security events, which also has an SC deployed on a private instance of Ethereum.

## 3. State of The Art

Several proposals have arisen in the last years aiming at protecting IoT ecosystems [24]. Thus, for instance, [40] proposes a security architecture that is based on the use of security events. Such architecture relies on a multi-relation between these attack-related elements: (i) security events categories, providing information about the impact of an attack over a given IoT device; (ii) vulnerabilities, to explain the causes of the attack; and (iii) attack surfaces, yielding information on how the attack was conducted.

On the other hand, several blockchain-based solutions have been proposed with the aim of leveraging the security attributes of the distributed ledgers within IoT scenarios. In this direction, the authors in [25] propose an IoT security framework for a smart home scenario. This framework applies a novel instance of blockchain by eliminating the concept of PoW and the need for coins. This work relies on a hierarchical structure that coordinates methods over the blockchain network to keep the security and privacy benefits offered by this technology. Such a hierarchical structure is more suitable for the specific requirements of IoT, since tasks on the network are performed in a different and adjusted manner than a common blockchain such as Bitcoin [26]. The framework proposes to manage the network, and the belonging devices with the methods store, access, monitor, genesis, and remove. This framework was exemplified in the context of a smart home, but the application is open to be used to other IoT contexts.

A blockchain-based framework to support access control in IoT is introduced in [27], implementing multiple smart contracts: (i) Access Control Contract (ACCs) to manage the authorization of users over an IoT device, next (ii) Judge Contract (JC) to implement a misbehavior-judging method to facilitate the dynamic validation of the ACCs, and (iii) Register Contract (RC) to register the information of the access control, and misbehavior-judging methods as well as their smart contracts. When an access request arrives to the framework, different validations are done with the smart contracts (i.e., ACC, RC, and JC) before resolving such request. The potentiality of the entire framework is demonstrated with a prototype implemented over a desktop computer, a laptop, and two Raspberry Pi 3.

In addition, [28] investigates the applicability of a blockchain to develop the next-generation SIEM 3.0 systems, designed to detect information security incidents in a modern and fully interconnected organization network environment. This work brings the next generation of SIEM to a qualitatively new and higher level by proposing a methodology for its evaluation based on the B method, the most popular formal method to be used in industry projects and safety-critical system applications [29]. This method allows for highly accurate expressions of the properties required by specifications and models systems in their environment.

Likewise, [30] shows a secure smart surveillance system based on microservices architecture and blockchain technology. This proposal uses microservices in order to decouple complicated video analysis functions and blockchain to keep the data in a safer state while implementing common surveillance functions, like real-time behavioral analysis of subjects, license plate recognition, face recognition, and gesture analysis over smart-contracts deployed in a blockchain network.

Another approach is proposed in [31], where a hybrid network architecture for a smart city is presented by leveraging the strength of emerging Software Defined Networks (SDNs) and blockchain technologies. This proposal presents a model based on two different groups: (i) the core network consisting of miners with high computational and storage resources and (ii) the edge network that is built with nodes with limited storage and computational capacity. The flow in this proposal works, as follows: IoT devices collect data from the real-world, then edge nodes filter the data, and core nodes perform the processing and analysis of information.

Another related work that is based on a smart home scenario is described in [32], where a proof-of-concept prototype is presented to simulate smart home applications, specifically, to measure humidity and temperature. In particular, authors leverage the use of blockchain to mine a smart-contract, which will be able to store both threshold and temperature values in the network that are collected every 15 s. When a measured value exceeds the threshold, a red LED will turn on, whereas a green LED will activate when the measured value is under the specified threshold.

Furthermore, in [33], the authors present a security solution called CitySense, a blockchain-oriented smart city solution. The proposal aims to monitor the quality of life within a smart city. Because IoT devices collect relevant data from the surroundings through their sensors, CitySense implements several types of smart contracts to receive and store data, which are analyzed under the Scrum methodology [34] to build smart services for citizens. Concretely, the Acquisition and Sorting Contract (ASC) receives measurements coming from IoT devices, validates where the measurement is coming from, and re-sends them, according to the location, to a specific and specialized smart contract, called a Geographic Contract (GC).

Additionally, a security proposal applying blockchain to smart cities is described in [1], which provides a secure communication platform for smart cities by integrating blockchain with smart devices. The proposed security framework is built on four layers: (i) Physical Layer, (ii) Communication Layer, (iii) Database Layer, and (iv) Interface Layer. Particularly, blockchain impacts the layers (ii) and (iii) by encrypting packages for the Communication Layer and storing records of packages in blocks for the Database Layer.

Another related work is presented in [35], which proposes BiiMED, as a blockchain framework for enhancing data interoperability and integrity regarding sharing of Electronic Health Records (EHR). That solution proposes the implementation of two components: (i) the Health Information System (HIS) and (ii) the BiiMED blockchain framework. The first component mainly provides a friendly interface to allow interaction with the blockchain framework, whereas the BiiMED component holds: (i) the Medical Facility access management contract, responsible for executing access control functions and (ii) the Trusted Third Party Auditor (TTPA) access management contract, responsible for validating the shared data and ensure its integrity.

For a quick reference, Table 1 shows a comparison of the previously analyzed proposals providing security for IoT environments while using blockchain. The comparison criteria were chosen based on the differences in the implementation strategy and the main purpose of each proposal.

As observed in Table 1, half of the related works integrate IoT scenarios, such as smart cities and blockchain, to tackle different security challenges. In particular, we found out that blockchain has been applied to support a variety of IoT operations like data synchronization, communication, or access control. In the paper at hand, we propose BlockSIEM, which, in contrast to all previous proposals, is specifically focused on the management of security events. Our proposal brings the main security features of blockchain to a regular SIEM to build a security solution that is specifically focused on IoT environments, in particular smart cities, being resilient, trust-oriented, auditable, and scalable. To the best of our knowledge, none of the presented security solutions applicable to smart cities holds those fundamental attributes with verifiable functionality.

## 4. BlockSIEM

This section describes the main aspects of BlockSIEM, our proposal for a blockchain-based and distributed SIEM to protect smart cities. BlockSIEM gathers security events coming from different IoT service providers, storing them in a distributed ledger of a blockchain that keeps them completely secure against any kind of unexpected modification. Additionally, our solution uses both internal and external threat intelligence to detect and prevent cyber attacks against the smart city services and intelligent devices, promptly warning about an in-progress attack. To this end, BlockSIEM satisfies the following goals:

- Resilient: in order to offer highly reliable security services, the solution should have the capability of ensuring protection of smart city services and attack detection, even if BlockSIEM is in a hostile situation.
- Trust-oriented: only trusted nodes belonging to the IoT service providers should be allowed to create transactions containing security events, avoiding any type of data pollution in BlockSIEM. In this sense, BlockSIEM features non-reputation and traceability.
- Auditable: BlockSIEM must be able to audit the block of security events to identify traces that refer to an incident response procedure, such as detecting the node that reported an event, the affected IoT devices, or discovering the causality relationship between events.
- Scalable: BlockSIEM should be able to effortlessly integrate new trusted nodes and miners into the blockchain network without impacting the operation adversely.

**Figure 1:**
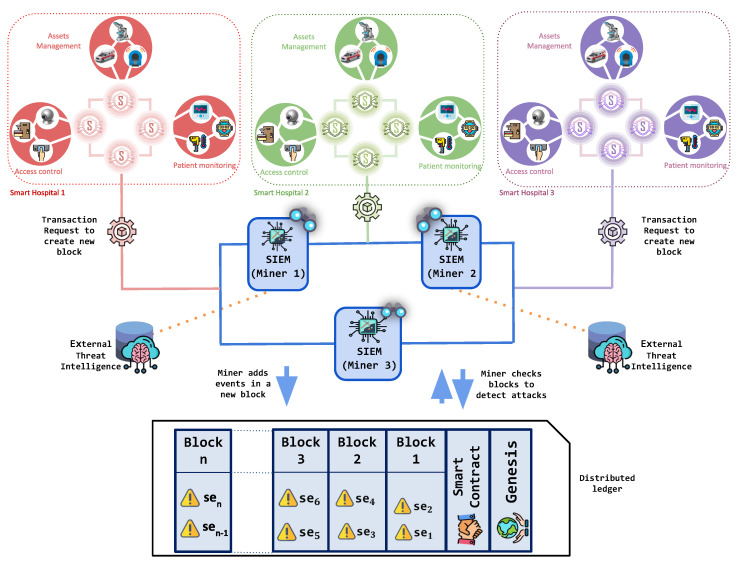
Architecture of a blockchain-based and distributed Security Information and Event Management (SIEM), BlockSIEM.

The architecture of BlockSIEM ={D,S,SG,M,T} is shown in Figure 1. Specifically, it encompasses the following elements: IoT devices (D), IoT sentinels (S), sentinels enabled as gateways (SG), SIEMs enabled as miners (M), and external Threat Intelligence providers (T). Each of these element acts in a different stage of the security events management process and is described in more detail next. Because BlockSIEM is based on blockchain technology, it is composed of *P2P network nodes*. In particular, BlockSIEM features two types of blockchain nodes, IoT sentinels and SIEMs enabled as miners.

### 4.1. Iot Devices

A Smart city involves the deployment of different services offered in scenarios, like smart hospitals, smart transportation systems, smart airports, and so forth [36]. Each scenario is supported by IoT devices ($D=\{D_{1},\ldots ,D_{n_{D}}\}$), which have the capacity of exchanging data with local or remote devices located in the Internet. IoT devices may be used for different purposes as they contribute to automated processes due to their capability to capture and process information in real-time [37]. Nonetheless, in some scenarios it is required to share information processed by IoT devices (e.g., sensed values) with a software or hardware component deployed in another scenario, which may use such information to trigger a process (e.g., activate an actuator).

In this context, IoT devices represent a vulnerable point of attack due to their massive presence in smart cities, but reduced ability to react against a cyber attack [38]. When considering the impact that a cyber attack would have on IoT devices and, consequently, on smart city services, one could easily say that these devices and their infrastructure should be protected. Moreover, the protection should guarantee that any attacker trying to make an unauthorized action over IoT devices may be correctly traced back.

### 4.2. Iot Sentinels

An IoT sentinel ($S_{i}\in S=\left\{S_{1},\ldots ,S_{n_{S}}\right\}$) is in charge of protecting a set of IoT devices ($D_{m}\subset D$) in its proximity against cyber attacks. Specifically, an IoT sentinel $S_{i}$, can detect attacks that are threatening the surrounding IoT devices $D=\{D_{1},\ldots ,D_{n_{D}}\}$ using multiple protection rings, as demonstrated in [24]. In this regard, whenever an intrusion attempt happens, an IoT sentinel ($S_{i}$) generates security events se and builds a transaction request that is sent to the set of SIEMs enabled as miners ($M_{n}\subset M$). Those SIEMs will mine the transaction and add it to the blockchain in the form of blocks. In the context of a smart city, different IoT sentinels ($S_{k}\subset S$) may be simultaneously deployed in one scenario in order to protect service-related IoT devices, depending on the number of devices and the generated network traffic.

A gateway strategy may be adopted, where only a chosen IoT sentinel reports security events to the set of SIEMs enabled as miners, in order to avoid multiple IoT sentinels generating transaction requests in the same scenario. By doing this, the IoT sentinel enabled as gateway ($SG_{i}\in \mathrm{SG}=\left\{SG_{1},\ldots ,SG_{n_{SG}}\right\}$) would gather all security events generated by other IoT sentinels. In this regard, one may naturally argue that a sentinel enabled as gateway ($SG_{i}$) should be endowed with top-level features to capture, store and share information, leading to expensive acquisition and maintenance costs. However, thanks to the benefits offered by the blockchain network, a sentinel enabled as gateway ($SG_{i}$) here is only required to gather and keep a small portion of security events (se) before creating an actual transaction. Thus, a sentinel enabled as gateway ($SG_{i}$) just needs to run a lightweight blockchain client, turning it into a new node of the blockchain. The lightweight blockchain client allows for a sentinel enabled as gateway ($SG_{i}$) to use the smart contract to format security events and add them to a transaction when a given threshold of events is reached. Likewise, a sentinel enabled as gateway ($SG_{i}$) can use the smart contract to delete specific security events from an already created transaction to avoid storing trash data in BlockSIEM. To avoid that the sentinel enabled as gateway ($SG_{i}$) becomes a single point of failure, it is important to implement an effective redundancy and availability strategy, such as allowing that other IoT sentinels ($S_{i}$) may eventually behave as a gateway or controlling the functionality of sentinels according to their reputation. An example of the last one is presented in [39], which proposes a CIDN (Collaborative Instruction Detection Network) with the capability of finding and avoiding false or bogus alarms that are based on a reputation model, which selects nodes with the best reputation according to its previous interactions, removing nodes that expose a malicious behavior.

### 4.3. Siems Enabled As Miners

A SIEM enabled as a miner ($M_{i}\in M=\left\{M_{1},\ldots ,M_{n_{M}}\right\}$) has mining and security functions. Regarding the mining functions, a SIEM enabled as a miner is in charge of receiving transactions requests, participating in the consensus algorithm and mining new blocks to be added to the blockchain. Therefore, a SIEM enabled as a miner ($M_{i}$) must have higher hardware capabilities to be able to solve a challenge in the blockchain network. In a deployment of such as SIEM in a smart city that is composed of multiple services, security events would be reported by a set of IoT sentinels enabled as gateways ($SG_{o}\subset \mathrm{SG}$). They generate multiple transaction requests that would be processed by a set of SIEMs enabled as miners ($M_{n}\subset M$), which would mine blocks to feed the blockchain. By leveraging the use of blockchain technology, the mined transactions acquire advantageous security attributes, e.g., traceability and non-repudiation, in addition to the traditional features of SIEM systems.

Regarding the security functions, a SIEM enabled as a miner ($M_{i}$) analyzes all of the security events contained in the distributed ledger of the blockchain with the purpose of detecting or preventing cyber-attacks. In order to do so, each SIEM enabled as a miner ($M_{i}$) has embedded internal Threat Intelligence which is composed of security policies, correlation rules, malware signatures, or any valuable information that allows the SIEM to prevent, detect, and respond to security incidents. Such internal Threat Intelligence is, in practice, a local database hosted by the SIEM and built through the experience of the local security team. When an attack is detected, different actions could be followed, depending on the type of incident and affected service. For example, a notification may be generated when a non-critical alarm is registered, or a sequence of batch commands may be launched over a controlled asset if a critical alarm is detected [38]. It is worth noting since each SIEM is fully synchronized on the blockchain current status, the mined events are simultaneously monitored by all of the miners. This feature permits, for example, to efficiently detect distributed attacks targeting different subsets of IoT devices.

It is worth mentioning that SIEMs enabled as miners could be hosted by different security providers at different levels, like (i) Internet Service Providers (ISP), which can be interested in providing security for residential customers, (ii) National Computer Emergency Response Teams (CERTs), monitoring security incidents with a possible massive impact, or (iii) Security vendors, which can offer IoT security protection under a specific subscription. In this context, even if all blockchain nodes are identified, not all nodes are necessarily trusted for sharing security events. Security events are fundamental to detect and prevent attacks through the use of Threat Intelligence

### 4.4. External Threat Intelligence

The external Threat Intelligence (T) is provided by a third-party service which is focused on detecting attacks against IoT infrastructures, such as, (i) identification of malicious websites being consumed by an IoT device, (ii) identification of bots attempting to connect to IoT devices, (iii) detection of spam data being transmitted between IoT devices, and (iv) recognition of advanced threats and malware campaigns, among others. In contrast with internal Threat Intelligence, the external one is commonly received in the way of feeds by subscription with some security provider, and it may be very valuable in the detection of attacks as security providers may have global visibility of incidents and use that information to warn their customer about potential attacks.

These detection tasks may conclude with the generation of Indicators of Compromise (IoC) and Indicators of Attack (IoA) that are used by BlockSIEM in the detection of an ongoing attack or to investigate a past attack sharing some common features with a known attack. External Threat Intelligence can also provide useful countermeasures for organizations to apply in the implementation of its cyber defense strategies, like Yara rules (https://github.com/Yara-Rules), correlation rules, security policies, and statistics model [40,41,42]. Intelligence information (security feeds) delivered by an external Threat Intelligence provider is useful for BlockSIEM, as it could use them to analyze security events that exist in the blockchain and, consequently, detect IoT attacks. BlockSIEM is also able to incorporate intelligence information from a third-party into its local Threat Intelligence database, so it can be usable later in the attack detection.

In addition, a smart city may be composed of several smart city scenarios, which share similarities, say, in the type of assets to protect. For example, a set of smart hospitals located in a smart city may be part of BlockSIEM, with each one configuring an IoT sentinel enabled as a gateway ($SG_{i}$) that reports security events. The reported security events from those similar scenarios may be extremely useful to gain insights about incidents per sector, to detect addressed attacks, and consequently to generate threat intelligence to protect specific assets. Other examples of smart city scenarios with some similarities that could also be integrated with BlockSIEM are set of governmental organizations or airports.

Thus, BlockSIEM may be seen as a security solution for smart cities that may gather security events coming from both similar and heterogeneous scenarios, which gives the opportunity to have a SIEM enabled as a miner with specific security functions per sector. In this case, each SIEM enabled as a miner ($M_{i}$) may be specialized in defending IoT devices from specific threats per sector, requiring to be connected to different per-sector Threat Intelligence providers. For example, a specific SIEM enabled as a miner ($M_{i}$) could consume feeds specialized in transport, health, or governmental sector, being reliable and efficient in each case.

## 5. Use Cases

In this section, we introduce five different use cases describing the main operations that the proposed BlockSIEM framework is able to carry out, namely: (i) adding blocks of security events to the blockchain, (ii) consuming the blockchain to detect distributed attacks, (iii) scaling up BlockSIEM, (iv) detecting attacks under hostile scenarios, and (v) auditing a security incident. Throughout this Section, we demonstrate that BlockSIEM can efficiently and effectively handle security events in each use case, preserving the blockchain’s relevant features.

### 5.1. Adding Blocks of Security Events to The Blockchain

As previously mentioned, IoT sentinels ($S_{k}\subset S$) are in charge of protecting IoT devices ($D_{m}\subset D$) in a specific scenario, like smart hospitals, smart transportation systems, smart airports, and so forth. IoT sentinels ($S_{k}\subset S$) are the only nodes in BlockSIEM that are able to create security events ($se_{m}$) whenever an unusual behavior is detected [24]. This action should only be guaranteed when IoT sentinels ($S_{k}\subset S$) are sufficiently reliable. Hence, the BlockSIEM implementation works strategically to control the information that is added to the blockchain, thus ensuring only the entry of reliable data.

Moreover, an IoT sentinel ($S_{i}$) can group security events in the same transaction in order to optimize the reporting of anomalies. This optimization generates a single block of clustered security events, which positively impacts the performance of the blockchain, as it avoids creating a block for each single event ($se_{i}$). To this end, the **Threshold of Security Events** ($\lambda_{se}>0$) is defined as the number of events that must be grouped to create a transaction and set in the configuration of each IoT sentinel ($S_{i}$).

Finally, when an IoT sentinel ($S_{i}$) creates a transaction request, it will be processed by a SIEM enabled as a miner ($M_{i}$), which is in charge of adding blocks to the blockchain. After a transaction is sent, the threshold of Security Events is reset to 0 to count another set of security events and generate a new transaction. Once the block is mined, all of the SIEMs enabled as miners (M) in BlockSIEM are synchronized and receive a copy of the block that was just mined, concluding the addition of security events to the blockchain. In the context of this research, the synchronization of the SIEMs enabled as miners (M) is achieved because of the underlying Ethereum protocol, which guarantees the successful functioning of this operation.

### 5.2. Consuming the Blockchain to Detect Distributed Attacks

The process of cyber-attack detection from the security events starts once a block with clustered security events is mined, and all of the SIEMs enabled as miners (M) of the blockchain become synchronized. Explicitly, once the block with security events has been successfully mined within the blockchain, each SIEM enabled as miner ($M_{i}$) checks the distributed ledger looking for potential events of interests. Here, each SIEM enabled as a miner ($M_{i}$) in the BlockSIEM framework uses the internal and external Threat Intelligence (T) to analyze the received information and leverage the security rules and policies to correlate the security events and, consequently, detect attacks against IoT devices. Once an intrusion detection has been carried out, the SIEMs enabled as miners (M) can counteract the ongoing threat by enforcing appropriate countermeasures.

A security solution, such as BlockSIEM raises awareness over the security events stemming from different scenarios, provides a great opportunity to detect distributed attacks. In a distributed attack, two or more IoT sentinels could notify different security events, which are apparently not related. However, the SIEMs enabled as miners would be able to correlate such security events and realize that they refer to the same security incident (e.g., they refer to the same threat source or the same attack technique). After a distributed attack is correctly detected, an alarm will be generated, which will be composed of individual security events.

**Figure 2:**
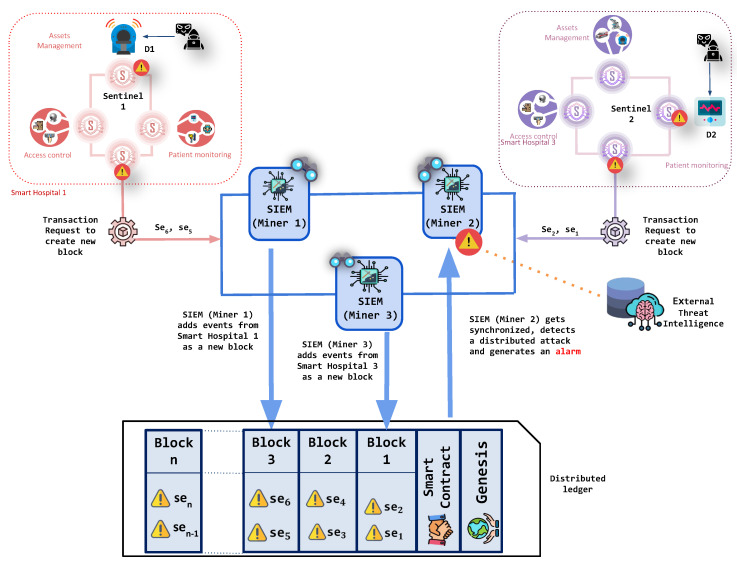
Detection of a distributed attack from security events coming from different sentinels.

Figure 2 illustrates this use case in BlockSIEM, where a distributed attack in a smart city is detected. It shows an attacker compromising an IoT device ($D_{1}$) that belongs to the smart hospital 1, which is protected by a sentinel ($S_{1}$). The attacker also jeopardizes an IoT device ($D_{2}$) in the smart hospital 3, which is monitored by another IoT sentinel ($S_{2}$). Each sentinel ($S_{1}$ and $S_{2}$) sends their corresponding security events toward the SIEMs enabled as miners (M) of the blockchain, which then process the transaction request and mine blocks of security events. Subsequently, the SIEMs enabled as miners (M) use external threat intelligence to detect the attack.

### 5.3. Scaling up BlockSIEM

By leveraging the scalability properties of the blockchain network, BlockSIEM allows for effortless integration of IoT sentinels as well as SIEMs enabled as miners. It is worth noting that each new node of the network (either an IoT sentinel or a SIEM enabled as a miner) must be authorized previously, before their actual functioning, to prevent potential impersonation attacks [43].

**Figure 3:**
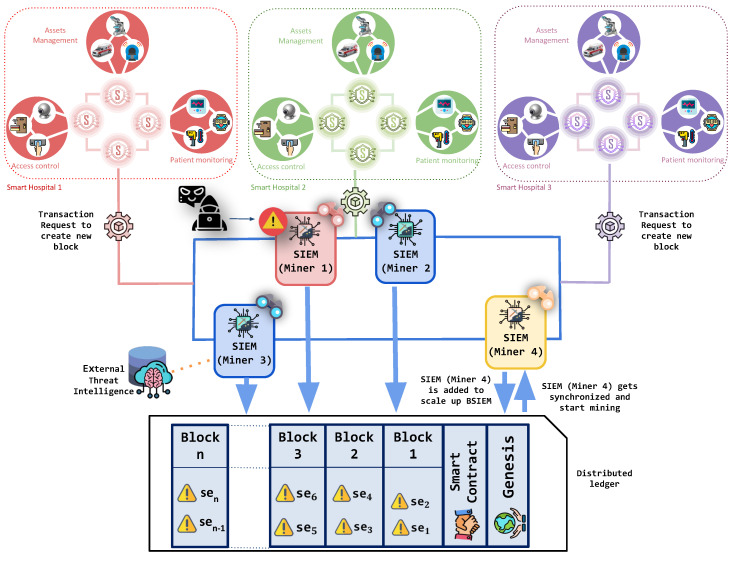
Reduction and increase of SIEMs enabled as miners in BlockSIEM.

Let us say that a set of SIEMs enabled as miners ($M_{1}$, $M_{2}$, and $M_{3}$), as shown in Figure 3, are the only nodes in BlockSIEM that are mining blocks. We want to enable a fourth miner within the P2P network to improve the overall performance of BlockSIEM. To do so, $M_{4}$ is added to the blockchain, which gets synchronized with the blocks of the BlockSIEM and includes the deployed SC. M4 starts mining smoothly due to BlockSIEM scalability property. Since the BlockSIEM network features a private blockchain, each node that would like to join must already be registered and known by the other nodes. Finally, all the SIEMs enabled as miners ($M_{1}$, $M_{2}$, $M_{3}$, and $M_{4}$) split the mining tasks between them, thus reducing each one’s single computational overhead.

### 5.4. Detecting Attacks under Hostile Scenarios

The BlockSIEM solution is resilient to unexpected situations, including attacks targeting the nodes that compose BlockSIEM without affecting security performance. This means that, if a SIEM enabled as a miner ($M_{i}$) is under attack, the IoT sentinels ($S_{k}$) will continue generating blocks of security events, and the remaining SIEMs enabled as miners ({M}$-M_{i}$) in the blockchain network will take care of mining transactions, so BlockSIEM operations are maintained.

Figure 3 shows a situation where SIEMs enabled as miners ($M_{1}$, $M_{2}$, $M_{3}$, and $M_{4}$) are mining blocks. $M_{1}$ fails down because of an attack. However, all other SIEMs enabled as miners ($M_{2}$, $M_{3}$, and $M_{4}$) continue mining all of the blocks. Hence, the attack does not affect the performance of BlockSIEM.

### 5.5. Auditing a Security Incident

As already mentioned, blockchain technology features several security properties, being non-repudiation and traceability among the most notable ones. Thanks to these properties, all registered information in BlockSIEM is permanently available and can be consumed in the future by any SIEM enabled as a miner ($M_{i}$).

Besides the security events, each block also contains metadata, such as the ID of the IoT sentinel ($Sentinel_{i}$) that created the security events, the creation date of the security events, and any other information that may be useful in the auditing process of a security incident. This approach allows BlockSIEM to guarantee a fully auditable system usable even for forensic processes.

The auditing of a security incident may start when a SIEM enabled as a miner ($M_{i}$) sets off an alarm. Then, the BlockSIEM administrator reviews the alarm details and starts a process of investigation and auditory to identify the source of the threat. The administrator inspects the security events that triggered the alarm and, additionally, reviews the sentinel(s) that reported the security events. The information about the IoT sentinel ID that detects the threat and reports the security events (together with all other available information regarding the security event report) may be consulted from the blockchain in a secure and trustworthy way, since such information is protected against modification, making it useful for forensic purposes.

## 6. Experiments

Several thorough experiments were conducted on the proposed solution to prove its suitability within a smart city ecosystem. Because BlockSIEM is composed of different elements, as shown in Figure 1, the experiments developed in this paper comprised the following infrastructure:

- **IoT sentinels ($S_{i}$)**: Each sentinel has been deployed on a Raspberry Pi 3 Model B, equipped with a quad-core 1.2GHz CPU, 1GB RAM, 16GB SD Card, and OS Ubuntu Mate 16.1.
- **SIEMs enabled as miners (**$M_{i}$**)**: Three identical virtual machines (VMs) have been deployed to host the SIEMs. Specifically, each VM features a total of 16 cores at 2.2 GHz and 16 GB of DDR4 memory at 2400 MHz. All SIEMs have been implemented using Alienvault OSSIM (https://www.alienvault.com/products/ossim) (Open Source SIEM) version 5.5.1 with an All-in-one installation.

For the ease of reading, the settings of the experiments are reported in Section 6.1, while a significant analysis of the results is carried out in Section 6.2.

### 6.1. Settings

The experiments were conducted by running one Ethereum [23] node on each physical component, i.e., the IoT sentinels and the SIEMs (miners). The SIEMs (miners) were able to create mined blocks thanks to their computational capabilities, whereas the IoT sentinels were only able to create transactions and send them to the correspondent SIEM.

**Figure 4:**
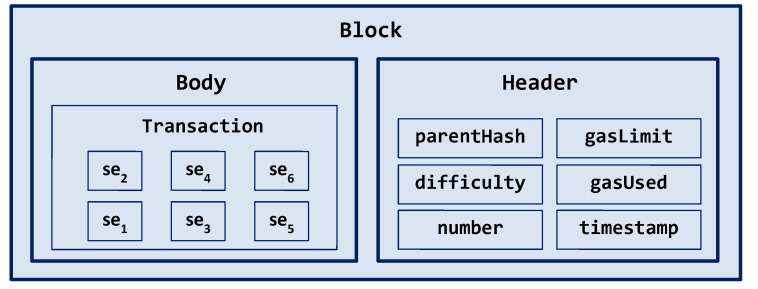
Block structure.

Each mined block in BlockSIEM is composed of a block header and a transaction, as illustrated in Figure 4. The header contains regular Ethereum header data (timestamp, difficulty, gas limit, uncles hash, gas used, among others), and the transaction includes the security events that were generated by IoT sentinels in the body field.

As mentioned in Section 5.1, BlockSIEM is based on a permissioned blockchain that only allows known nodes (IoT sentinels and SIEMs enabled as miners) to be part of the network. It is worth mentioning that the component nodes have been set as *static nodes*, disabling the discovery functionality of Ethereum. The consensus mechanism was the one currently supported by Ethereum, i.e., PoW; however, as Ethereum evolves, a more efficient consensus mechanism, e.g., PoS, could be used instead, reducing the time and effort that are currently required for the mining process.

Blockchain generally uses a reward system to encourage the participation of the nodes that mine the blocks, being the Ether in a public blockchain network the token paid to the miner nodes. The reward system of BlockSIEM defines its own token, similar to Ether, but only valid internally. In a real scenario, users interested in protecting their own IoT devices could host an IoT sentinel connected to BlockSIEM to share security events.

**Figure 5:**
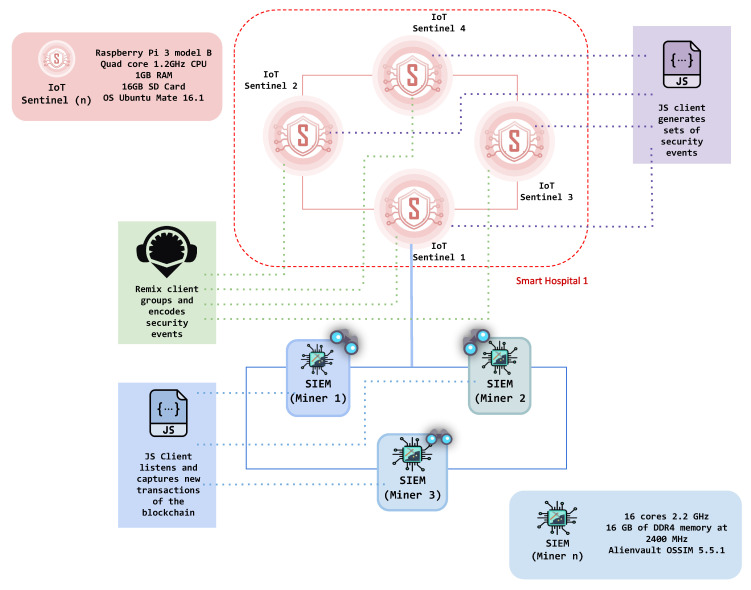
Scenario for the execution of experiments.

The experiments were carried out using several clients that ease the implementation of BlockSIEM, namely: (i) A Remix (https://remix.ethereum.org/) client for the IoT sentinel, which groupes and encodes security events to be added to a new transaction, (ii) a JavaScript (JS) client for the SIEM (miner), running in the desktop computer, and responsible for listening and capturing new transactions of the blockchain, in order to decode security events and make them understandable for the OSSIM server, and (iii) a JavaScript (JS) client, running in Raspberry Pi and emulating the monitoring action that an IoT sentinel performs to generate a set of security events. An illustration of the scenario prepared for the experiments may be found in Figure 5.

### 6.2. Analysis Of Results

This Section offers an in-depth analysis of the outcomes from the experiments that were conducted over the BlockSIEM. The obtained results will be organized around two types of metrics (performance and blockchain, respectively), as shown in Table 2.

As observed in Table 2, metrics from the performance category refer to the computational performance of BlockSIEM which focus on the consumption of resources for the considered use cases. On the hand, metrics of the blockchain category refer to the own behavior of BlockSIEM in terms of number of blocks and the number of events.

To validate the capabilities of BlockSIEM, three scenarios have been considered and tested, which are strongly connected to the use cases presented in Section 5:

1. Adding blocks of security events to the blockchain: IoT sentinels communicate or send security events to the distributed SIEMs using different thresholds of security events $\lambda_{se}$
2. Consuming the blockchain to detect distributed attacks: the distributed SIEMs use correlation rules to detect distributed attacks stemming from the received Sentinels’ events.
3. Detecting attacks under hostile scenarios-Scaling up BlockSIEM: even in adverse scenarios, BlockSIEM is capable of offering beneficial blockchain services, relying on SIEMs failures and subsequent reactivations.

Note that, during the experiments, all if the metrics have been measured over the SIEMs (miners) through a python script set on a dedicated CPU to avoid conflicts due to possible context switches. In particular, one of the cores of the VM hosting the SIEM has been assigned to the measuring python script to avoid that, during the experiments runs, other processes may interfere by asking for the same CPU and, thus, generating a context switch. Such phenomenon may adulterate the correctness of the results.

#### 6.2.1. Scenario 1: Adding Blocks of Security Events to The Blockchain

In this experiment, the IoT Sentinels were set up in order to generate and send events to the three SIEMs enabled as miners with a fixed frequency (i.e., every 5 s). This regular generation of security events was selected for the experiments since it represents the prevailing state of operation of BlockSIEM where the integrity of security events is critical to detect complex attacks like Advanced Persistent Threats (APT). In those cases, the external and internal threat intelligence is fundamental to warn the SIEM administrator about such anomalous behaviors (within an apparent normality), which otherwise would not be easily noted in a manual inspection. On the other hand, situations with burst of security events would not actually require advanced threat intelligence, because bursts would be easily noticeable by the SIEM administrator. However, in this last situation, our proposal (BlockSIEM) would be able to protect security events in terms of integrity and non-repudiation too. Specifically, two rounds of execution were performed as follows:

1. Round 1: no critical security events (e.g., informational Syslog messages) are communicated from the IoT sentinel to the SIEMs enabled as miners, which can be retained in the sentinel until reaching a threshold of security events ($\lambda_{se}=5$), and then be grouped in one transaction. Thus, a transaction is generated every 30 s.
2. Round 2: critical security events (e.g., emergency Syslog messages) need to be communicated in a short time from the IoT sentinel to the SIEMs enabled as miners, incorporating 1 security event per transaction ($\lambda_{se}=1$). Thus, a transaction is generated every 5 s.

**Figure 6:**
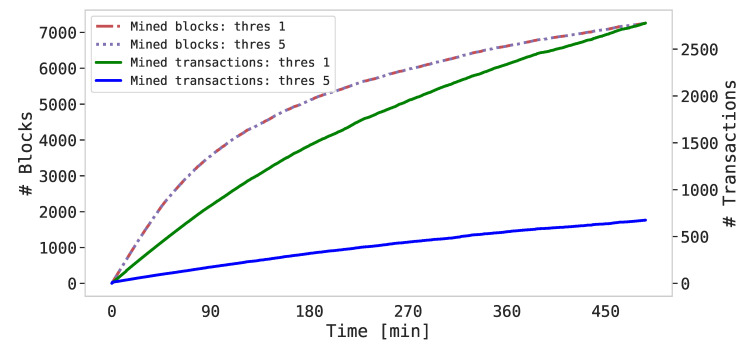
Mined blocks and transactions of the SIEMs enabled as miners with different thresholds.

The combined outcome of this experiment (rounds 1 and 2) is depicted in Figure 6. In both cases, the time execution was about 8 h for each round, so we decided to combine both of them in a single graphical representation. Specifically, Figure 6 compares the trend of the blocks and the progression of the transactions mined by the SIEMs enabled as miners with both thresholds. It is worth noticing here the difference between block and transaction: that is, even if no security events are generated by the Sentinels, the SIEMs continue to mine blocks in order to maintain the correct operation of the blockchain following the Ethereum protocol. Those blocks (e.g., communication and/or synchronization blocks) contain an empty data field. On the other side, instead, the transactions are generated by the Sentinels and contain data that are related to the detected intrusion(s). Note that Figure 6 has been depicted with a double y-axis.

A salient aspect in Figure 6 is represented by the curves’ shapes. That is, the trend of the mined blocks is exponential, since the mining process for the SIEMs becomes computationally more expensive as the number of blocks of the blockchain increases (i.e., the mathematical challenge for the miners is harder to solve). Surprisingly, this trend is independent from the assigned threshold, so the number of blocks remains constant over a fixed time window. Oppositely, the trend of the curves representing the mined transactions is quite linear since, as already mentioned, the frequency of the generated Sentinels’ events is fixed. Consequently, due to the different thresholds, the number of total transactions mined is distinct, i.e., almost 3000 transactions for $\lambda_{se}=1$ and 700 for $\lambda_{se}=5$. As for the mined block curve, transaction curves are also affected by the increasing complexity, so they lose their linearity in the last part of the experiment (i.e., starting from 6 h of run duration).

All in all, the findings of this experiment demonstrated that the proposed BlockSIEM framework is able to manage a high number of events during a considerable time window, preserving the beneficial features offered by the blockchain technology.

#### 6.2.2. Scenario 2: Consuming the Blockchain to Detect Distributed Attacks

In this experiment, the main objective is to test the capabilities of the BlockSIEM framework to detect distributed attacks and, consequently, to enforce the appropriate reaction. The procedure executed during this experiment is reported in Algorithm 1.

In detail, Algorithm 1 requires one or more Sentinels, one or more Miners, a threshold of security events $\lambda_{se}$ set, and a certain quantity of SIEM directives containing the correlation rules and actions. When the experiments starts, the Sentinels, which are in charge of monitoring the surrounding IoT devices, generate security events containing data referring to possible threats (lines 1–3). Then, when the number of generated events is equal to the assigned threshold $\lambda_{se}$(line 4), a transaction is sent to the corresponding miner (i.e., SIEM, line 5), which we assume to be fixed for each Sentinel. Based on the Ethereum protocol, one of the miner nodes within the Ethereum private network mines the transaction into the blockchain, spending a certain amount of time which is measured (lines 9–10). Once the transaction is effectively mined, the miners are in charge of consuming such transaction in order to incorporate it into their local database of security events (lines 11–12). If during the incorporation process a correlation rule inside a directive is triggered, then the miner automatically fires an action $\delta_{i}$ (lines 13–14). The time spent between the incorporation into the local database and the fired action, which is referred to as response time, is also measured (line 15).
**Algorithm 1** Scenario 2 Experiment$\left(\lambda_{se},S,M,\Delta ,A\right)$.**Require:**S,M≠null                 ▹ Number of Sentinels and Miners **Require:**$|\lambda_{se}|>0$              ▹ Threshold for security events per transaction **Require:**|Δ|>0             ▹ SIEM directives containing the correlation rules **Require:**|A|>0                  ▹ SIEM actions when a rule is triggered 1:**for all**s∈S**do**                   ▹ Sentinels monitoring IoT devices2:    Generate security event $se_{i}\in \mathrm{SE}$3:    $X\leftarrow se_{i}$                       ▹ Accumulate security events4:    **if** $\left|X\right|=\lambda_{se}$**then**                       ▹ Threshold is reached5:        Send transaction to the corresponding miner m∈M6:        X←∅                          ▹ Empty the accumulator7:    **end if**8:**end for**9:Transaction is eventually mined by a miner m∈M     ▹ Based on Ethereum protocol10:Measure mining time of M11:**for all**m∈M**do**                  ▹ SIEMs consuming the blockchain12:    Incorporate the blockchain event in the local DB13:    **if** $\delta_{i}\in \Delta$ is activated **then**                ▹ Correlation rule triggered14:        Enforce the action $a_{i}\in A$15:        Measure the response time16:    **end if**17:**end for**


**Figure 7:**
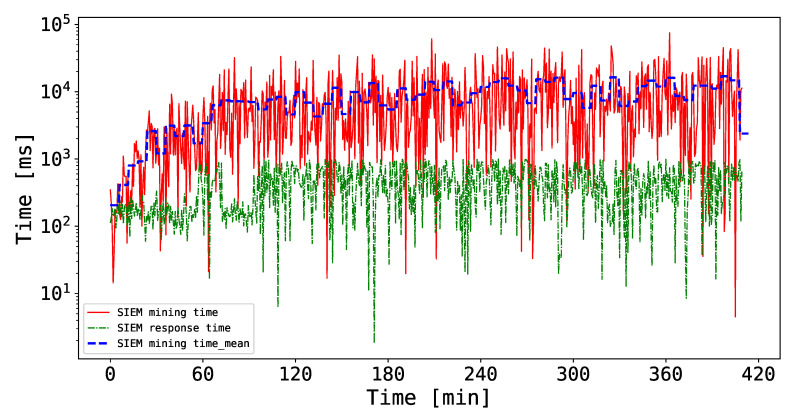
SIEM mining and response times in logarithmic scale.

Thus, the results of these experiments are shown in Figure 7. Note that, in this case, the duration of the experiment is about 7 h, and the chosen threshold $\lambda_{se}=5$. In particular, the trends of the SIEM mining time and response time during the chosen time window is plotted in logarithmic scale. It is worth mentioning that the depicted time measurements are the arithmetic means of the three miners measured time. To this extent, it is possible to notice that the mining time increases over time, spanning from a few milliseconds to 70 s. The main reason behind this outcome is the increasing mining complexity which forces the miners to solve computationally expensive mathematical challenges to mine a block. Nonetheless, the mean of the SIEM mining time shown in Figure 7 sticks between 1 and 10 s, which one could argue to be reasonable for the proposed scenario. Regarding the SIEM response time, instead, the curve floats between a hundred and a thousand milliseconds, which is more than acceptable when considering the computational effort required for the SIEMs to perform their duties together with the blockchain-related processes.

Overall, the results of this experiment show that the proposed BlockSIEM framework is capable of managing the security events coming from different data sources (from the reception to the reaction) in an acceptable time, even in a stressed scenario. In this sense, BlockSIEM is able to handle distributed attack scenarios.

#### 6.2.3. Scenario 3: Detecting Attacks under Hostile Scenarios-Scaling up BlockSIEM

Throughout this experiment, our goal is to test the reliability of the BlockSIEM framework against possible adverse situations (i.e., those in which the SIEMs are the target(s) of an attack) and subsequent SIEM reactivations, which simulates the joining of a miner within the Ethereum private network. More specifically, the SIEM mining processes were shut down for a fixed time (i.e., about 10 min), and then switched on. In literature, a plethora of possible attacks targeting critical monitoring infrastructures have been analyzed. The main objective of those attacks is to threaten the availability of the monitoring services (in our case, the SIEMs enabled as miners). In the proposed experiment, we decided to simulate a quite effective attack that is able to completely stop the SIEMs services, which are later reactivated. In this sense, we believe that we chose to simulate the worst scenario, since the effectiveness of such attacks in real conditions may be considerably lower, e.g., compromising a single service and not the entire system. The total duration of the experiment was almost 6 h.

**Figure 8:**
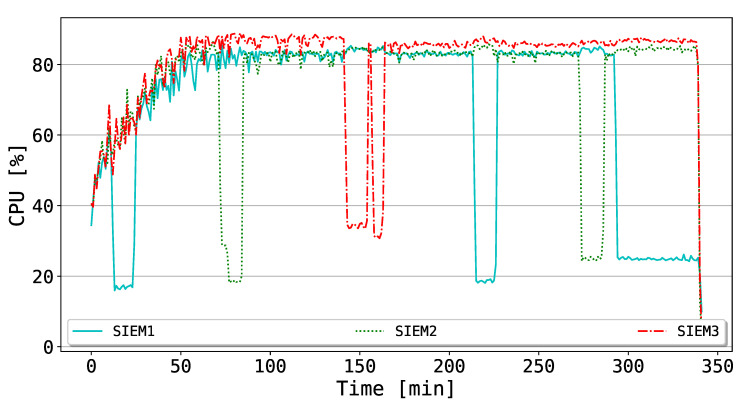
CPU utilization for the different SIEMs during drops and successive reactivations.

In Figure 8, the CPU consumption of the SIEMs is depicted. Globally, the CPUs curves of the SIEMs reach stability after the first increasing trend (i.e., around 50 min from the beginning of the experiment). Subsequently, they are constantly above 80% of utilization, that is, they are highly stimulated in order to mine new blocks. Such an outcome may be explained by looking at the detail of the Ethereum protocol: in fact, the version of *geth* (https://geth.ethereum.org/) used in the performed tests leverages only the CPU mining, without considering the possibility of performing GPU mining. To this extent, one could easily argue that GPU features the most appropriate hardware components to mine blocks into the blockchain, since they are optimized to compute mathematical operations. Additionally, it is possible to observe that, according to the alternating deactivations of the SIEM mining processes, the affected SIEM significantly decreases the CPU utilization, while the other SIEMs need to further stress their computational capabilities, even if the spike is not remarkable. Later, when the mining process for the dormant SIEM is reactivated, the trend for all the miners goes back again to a stable value.

**Figure 9:**
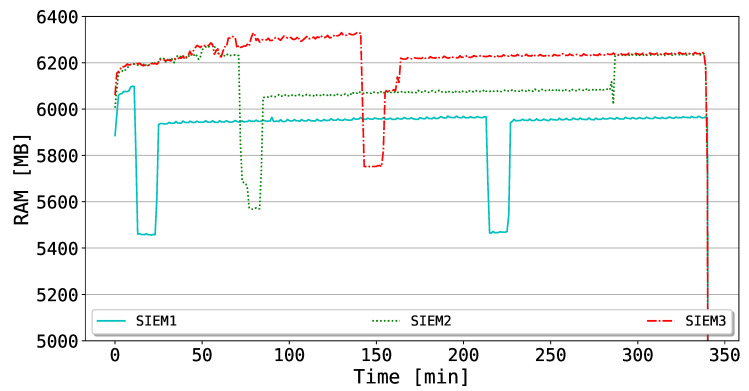
RAM utilization for the different SIEMs during drops and successive reactivations.

Moreover, in Figure 9, the RAM utilization of the SIEMs is shown. As in the case of CPU consumption, the RAM of the corresponding SIEM is also affected by the drop of the mining process, which costs a negative spike of around 600 MB. After the reactivation, the RAM values turn back to a stable state. Different from the CPU utilization of Figure 8, the RAM consumption does not present any additional spike for the other SIEMs linked to the deactivation of the affected one. This evidence is also justifiable by considering the Ethereum CPU mining, which does not affect the RAM consumption once the private network is up.

All in all, the outcomes of this experiment indicate that the designed BlockSIEM framework is reliable against possible adverse situations, in which the SIEMs are targets of attack and, thus, are not able to provide their services. Thanks to the reliability of the blockchain protocol of the private network, the unaffected nodes can effortlessly manage the computational overload.

Currently, Ethereum uses Proof of Work (PoW) as the consensus mechanism which is dependable on the number of nodes that accept a transaction. This consensus mechanism is vulnerable to situations where the majority of nodes get infected, since such compromised nodes could approve a forged or invalid transaction. The aforementioned situation may provoke that data stored in the blockchain loses its integrity and non-repudiation attributes. Even if experiments were implemented in Ethereum, our proposal BlockSIEM is agnostic of the consensus mechanism, allowing the use of another mechanism such as PoS, PoI, or PoA as long as the blockchain framework supports it.

## 7. Conclusions and Future Work

Smart cities are increasingly becoming a reality nowadays, and their advanced services towards citizens often rely on IoT devices. Unfortunately, such IoT devices, are frequently poorly secured, leading to an optimal playground for cyber criminals, constituting a non-neglectable risk for the wide deployment and success of such Smart cities.

By leveraging the benefits of blockchains, this paper presented BlockSIEM as a tool to contribute directly to the safety of IoT ecosystems by strictly managing the security events stemming from the IoT Sentinels shielding a given group of IoT devices and therefore preserving integrity and non-repudiation. Additionally, BlockSIEM offers desirable features for a sturdy security system such as resilience, trust-orientation, auditability and scalability. Thoroughly conducted experiments have shown that BlockSIEM performs desirably with low transaction times, being mainly affected by the Threshold of Security Events ($\lambda_{se}$) and the consensus method.

As for future works, we plan to allow for new types of transactions in our solution according to the type of security event detected by the IoT sentinel, bypassing the threshold ($\lambda_{se}$), e.g., more critical security events could be added to the blockchain with a higher priority, whereas medium or low priority events could wait to be grouped. Finally, we will study the feasibility of building a new generation of IoT devices that can be blockchain-capable, qualified to report internal security events to the blockchain.

## Figures and Tables

**Table 1:** Comparative table of the analyzed related works.

Related Work	Blockchain Type	Consensus Algorithm	Blockchain Platform	Asset to Protect	Use-Case Scenario
Dorri et al. [25]	Permissioned	N/A	N/A	Policies and transactions	Smart Home
Zhang et al. [27]	N/A	PoW	Ethereum	Access control policies	N/A
Miloslavskaya et al. [28]	Permissioned	N/A	N/A	Security events	Smart Office/ Organization
Nagothu et al. [30]	N/A	PoW	N/A	Video analysis data	Smart City
Sharma et al. [31]	Permissioned	PoW	Ethereum	General data	Smart City
Xu et al. [32]	Permissioned	PoW	Ethereum	Temperature and humidity measurements	Smart Home
Ibba et al. [33]	Permissionless	PoW	Ethereum	Environmental measurements	Smart City
Biswas et al. [1]	Permissioned	N/A	N/A	Data communication (messages)	Smart City
Jabbar et al. [35]	Permissioned	N/A	Ethereum	Electronic Health Records (EHR)	Healthcare Field
Pardo et al. [12]	Permissioned	PoW	Ethereum	Security events	Smart Home

**Table 2:** Performance and blockchain metrics for BlockSIEM.

Category	Name	Description
*Performance*	CPU	SIEM (miner) CPU usage along an experiment time lapse
	RAM	SIEM (miner) RAM usage along an experiment time lapse
*Blockchain*	Number of blocks	Blocks added to the blockchain
	Number of events	Security events added to the blockchain
